# School Leadership and Cyberbullying—A Multilevel Analysis

**DOI:** 10.3390/ijerph14101226

**Published:** 2017-10-15

**Authors:** Sara B. Låftman, Viveca Östberg, Bitte Modin

**Affiliations:** Centre for Health Equity Studies (CHESS), Stockholm University/Karolinska Institutet, 10691 Stockholm, Sweden; viveca.ostberg@chess.su.se (V.Ö.); bitte.modin@chess.su.se (B.M.)

**Keywords:** cyberbullying victimization, cyberbullying perpetration, cyber harassment, school climate, students, contextual

## Abstract

Cyberbullying is a relatively new form of bullying, with both similarities and differences to traditional bullying. While earlier research has examined associations between school-contextual characteristics and traditional bullying, fewer studies have focused on the links to students’ involvement in cyberbullying behavior. The aim of the present study is to assess whether school-contextual conditions in terms of teachers’ ratings of the school leadership are associated with the occurrence of cyberbullying victimization and perpetration among students. The data are derived from two separate data collections performed in 2016: The Stockholm School Survey conducted among students in the second grade of upper secondary school (ages 17–18 years) in Stockholm municipality, and the Stockholm Teacher Survey which was carried out among teachers in the same schools. The data include information from 6067 students distributed across 58 schools, linked with school-contextual information based on reports from 1251 teachers. Cyberbullying victimization and perpetration are measured by students’ self-reports. Teachers’ ratings of the school leadership are captured by an index based on 10 items; the mean value of this index was aggregated to the school level. Results from binary logistic multilevel regression models show that high teacher ratings of the school leadership are associated with less cyberbullying victimization and perpetration. We conclude that a strong school leadership potentially prevents cyberbullying behavior among students.

## 1. Introduction

Cyberbullying can be defined as “the use of electronic communication technologies to bully others.” [1] (p. 1074). Traditional school bullying has been defined in terms of negative acts committed towards an individual by one or more other persons [2]. Three common criteria in the definition of traditional forms of school bullying are repetition, unequal power, and intentional harm [3]. Traditional school bullying includes negative actions that are most often performed face-to-face, such as showing disapproval, teasing, ostracism, and physical harm. Cyberbullying concerns such negative actions that are performed via mobile phones, computers, and other electronic devices. While cyberbullying shares attributes with traditional forms of school bullying, it also presents itself with some unique features. For instance, cyberbullying can occur anywhere and anytime, the “audience” is undefined and can potentially be much larger than in the case of traditional bullying, and cyberbullying perpetrators have, a least in theory, greater opportunities to be anonymous than do perpetrators of traditional bullying [4]. While repetition is a core element in the definition of traditional school bullying, an act of cyberbullying can be performed only once but be digitally repeated over and over again through e.g., the sharing of images.

Earlier studies have shown that students’ involvement in cyberbullying and traditional bullying to a great extent overlaps [1,4,5,6,7,8]. Some scholars argue that cyberbullying creates very few new victims, implying that it is merely another means of harassing those who are already exposed to traditional forms of bullying [9]. Yet, cyberbullying has demonstrated independent associations with students’ psychological health outcomes also when adjusting for traditional forms of bullying [4,8,10,11,12], indicating that cyberbullying behavior is (at least to some extent) a distinct phenomenon that is relevant to study in itself.

A body of research has examined associations between various school-contextual characteristics and traditional forms of bullying. Traditional bullying has been shown to be less frequent in schools characterized by features such as clear rules, positive student-teacher relations, and teachers’ disapproving attitudes towards bullying and their intervening against bullying when it occurs [13,14,15,16]. Another school-contextual characteristic that is negatively related to bullying is collective efficacy, i.e., the degree of shared trust and support [17]. Aspects of schools’ professional cultures in terms of the school leadership, teacher affiliation, and collaborative activities have also demonstrated associations with traditional bullying behavior. Roland and Galloway [18] and Ertesvåg and Roland [19] used Norwegian data collected from both teachers and students, and found that schools in which the teachers rated the school leadership, teacher affiliation, and collaborative activities high had lower rates of traditional bullying victimization and perpetration. In a recent study of English schools, Bevilacqua et al. [20] used student survey data combined with school-level information including ratings of the school quality by the Office for Standards in Education, Children’s Services and Skills (Ofsted), which is a governmental authority that carries out inspections of schools in England. Students in schools which had been rated as “Good” were shown to be more likely to report traditional bullying victimization compared to those in schools categorized as “Outstanding”. The authors interpreted the finding by proposing that a highly qualified school leadership evokes a school climate characterized by disapproval of bullying, but acknowledged that also other aspects of school quality such as school ethos and awareness of bullying behavior may be of importance [20]. Another recent English study investigated school characteristics derived from Ofsted and from a teacher survey, and links with bullying victimization and perpetration [21]. The results showed that the quality of the leadership and management was associated with the occurrence of bullying victimization and perpetration, but that school policies, in particular those related to bullying, contributed more to explaining the variation in victimization and perpetration across schools [21].

Some studies have investigated the links between school-contextual characteristics and students’ involvement in cyberbullying behavior specifically. A negative school climate has been shown to be associated with both more cyberbullying victimization and more cyberbullying perpetration, albeit with relatively small effect sizes [22]. A study of Finnish data reported that students’ perception of their teachers’ abilities to intervene against bullying was associated with more cyberbullying perpetration [23]. The authors interpreted this result in terms of students tending to drift into cyberbullying rather than traditional bullying perpetration because it is less overt [23]. Bevilacqua et al. [20] analyzed not only traditional but also cyberbullying behavior and found that in schools with poor quality (i.e., classified as “Requires improvement”), cyberbullying perpetration was more common than in schools rated as “Outstanding”. No association was however found between school quality and cyberbullying victimization. Still, the finding that cyberbullying perpetration was less common in the schools with the highest quality suggests that a strong school leadership, being one aspect included in the “school quality” measure, may prevent against cyberbullying behavior among students. Yet, to the best of our knowledge, there are hitherto no studies that have investigated the links between teachers’ ratings of the school leadership and student-reported cyberbullying behavior.

The current study focuses on cyberbullying behavior among students in Sweden, a country which in international comparison has low rates of bullying at school, but also relatively low rates of cyberbullying [24]. For instance, in the international Health Behavior in School-aged Children (HBSC) study of 2013/14, 6% of 15-year-old Swedish students reported to have been cyberbullied by messages at least once (the HBSC average was 11%), and 6% reported to have been cyberbullied by pictures at least once (the HBSC average was 9%) [24]. Using new Swedish data that combines information from two surveys collected amongst teachers and amongst students, respectively, the aim of the current study is to assess whether teachers’ ratings of the school leadership, as one specific aspect of the school context, are associated with the occurrence of cyberbullying victimization and perpetration among upper secondary students. We hypothesize that strong school leadership, as assessed by the teachers, is associated with less cyberbullying victimization and perpetration as reported by students.

## 2. Materials and Methods

### 2.1. Data and Procedure

Data from two separate surveys performed in the spring of 2016 were combined: the Stockholm School Survey (SSS) and the Stockholm Teacher Survey (STS). The SSS is a cross-sectional survey conducted biennually by Stockholm Municipality among students in grade 9 of the compulsory school and grade 2 of the upper secondary school, in all public schools and in a number of independent schools in Stockholm. The questionnaires are completed by the students in the classroom and recollected by the teacher. The survey includes questions on alcohol use, drug use, smoking, and criminal behavior, but also covers topics such as social relations and the situation at school [4,16,25]. In the SSS of 2016, external non-response has been estimated to approximately 22% [26]. The STS was carried out in 2014 among senior-level teachers in the schools participating in the SSS, and in 2016 among senior-level teachers as well as among upper secondary school teachers in all the schools participating in the SSS. The main purpose of the STS was to collect information about school characteristics in terms of teachers’ ratings of—for example—the school leadership, cooperation and consensus, and school ethos but also about teachers’ working conditions, in order to study whether and how these aspects were related to student-reported outcomes in terms of psychological health, bullying, and academic achievement. The STS was performed though a web-based questionnaire. The response rate was 58% among upper secondary school teachers. To construct school-level measures, teachers’ ratings were aggregated to the school level, which were linked to the student-level data from the SSS.

In the present study, we use data from the SSS of 2016 collected among students in the second grade of upper secondary school (17–18 years) with matched school-level information from upper secondary school teachers participating in the STS of 2016. The matched data set includes information from 6129 upper secondary school students and 1251 teachers in 58 schools. The minimum number of students in a school was 10 and the maximum number was 362. For our study sample, we excluded 62 students with internal non-response on migration background, resulting in a study sample of 6067 students distributed across 58 schools. Due to item non-response also on our dependent variables, the analyses are based on *n* = 5657 (cyberbullying victimization) and *n* = 5781 (cyberbullying perpetration), corresponding to 92.2% and 94.3% of the responding 6129 students respectively.

### 2.2. Variables

Cyberbullying behavior was captured by questions on cyberbullying victimization and perpetration posed to students in the SSS. These questions were asked immediately after a set of questions on traditional bullying behavior. The opening question of the battery concerned whether the student had been bullied or harassed at school this school year, and response categories included a range of (non-mutually exclusive) items on various forms of traditional bullying victimization (e.g., whether the student had been teased, ostrasized, physically hurt, and whether someone had spread rumours). Thus, in the subsequent questions, students were probed with examples of the practical meaning of “bullied or harassed”.

Cyberbullying victimization was measured by the question: “Have you been bullied or harassed on the Internet or by text messaging (SMS/MMS) this school year?” The response categories were “Yes”, “No”, and “Don’t know” where the last category was coded as missing.

Cyberbullying perpetratation was measured by the question “Have you taken part in the bullying or harassment of other students on the Internet or by text messaging this school year?” Again, the response categories were “Yes”, “No”, and “Don’t know”, where the last alternative was coded as missing.

School leadership was captured by teachers’ responses in the STS, and measured by an index constructed from ten items. The items were: (a) “The management has an interest in pedagogical questions”; (b) “The management shows an understanding of my work problems”; (c) “The school leaders have high expectations of me as a teacher”; (d) “When the management makes decisions on important issues they first discuss it with the teaching staff”; (e) “The majority of teachers’ understanding of school goals and policies align with the management’s”; (f) “The management allows room for teachers’ pedagogical freedom”; (g) “I regularly receive feedback from the management about my performance as a teacher”; (h) “The management is a good support for teachers experiencing difficulties with a class”; (i) “The distribution of responsibility between teachers is clear at this school”; and (j) “This school is led in a good way”. The response categories were: “Strongly agree” (5); “Agree” (4); “Neither agree nor disagree” (3); “Disagree” (2); and “Strongly disagree” (1). Values from all items were added, thus forming a scale ranging 10–50 with higher values indicating higher teacher ratings of the school leadership. The measure was based on exploratory and confirmatory factor analysis and demonstrated good psychometric properties (RMSEA = 0.061; CFI = 0.993; TLI = 0.990). The index also shows high internal consistency (Cronbach’s alpha = 0.90). To capture teachers’ ratings of the school leadership at the school-level, we used the mean value of the school leadership index for each school. In order to detect potentially non-linear associations, we divided our study sample into three categories of about equal size, in order to classify students into schools with relatively weak, intermediate, and strong school leadership.

A set of control variables were included.

Gender was measured by the question “Are you a boy or a girl?” and the response categories “Boy” and “Girl”. Students who did not respond to this question (3.4%) were kept as a separate category.

Family structure was captured by the question “Which people do you live with?” with a list of boxes to be ticked. Those who ticked “Mother” and “Father” were classified as living with two parents in one household and were contrasted against all others.

Parents’ university education was assessed through the question “What is the highest education your parents have?” The response categories, to be ticked for mother and father separately, were: “Old elementary school (folkskola) or compulsory school (max 9 years schooling)”, “Upper secondary school”, “University and university college”, and “Don’t know”. Those who ticked “University and university college” for at least one parent were classified as having one parent with university education, and were contrasted against all others.

Migration background was measured by the question “How long have you lived in Sweden?” The response categories were: “All my life”, “10 years or more”, “5–9 years”, and “Less than 5 years”. The last two categories were merged due to small numbers.

### 2.3. Ethics

Data from the Stockholm School Survey are filled in anonymously (with no information on personal identification) and are therefore not subject to consideration for ethical approval, according to a decision by the Regional Ethical Review Board of Stockholm (2010/241-31/5). Ethical permission has been approved for the Stockholm Teacher Survey (2015/1827-31/5).

### 2.4. Statistical Method

Since the data structure is hierarchical with students nested within schools, and since school leadership, which is our main independent variable of interest, is measured at the school-level, multilevel analysis was applied. Two-level binary logistic regression models were estimated in Stata 13 using the “xtmelogit” command.

## 3. Results

Descriptive statistics of the study sample are presented in Table 1. Among the students, 7.3% reported to have been subjected to cyberbullying victimization, and 3.0% to have been involved in cyberbullying perpetration during the current school year.

Next, descriptive results are presented by categories of teachers’ ratings of the school leadership (Table 2). To the right of the columns displaying the numbers of students and schools in each category, mean values and ranges of teachers’ ratings of the school leadership are presented. The next two columns show the percentages of students who report cyberbullying victimization and perpetration by the three categories of teacher-rated school leadership. Clear gradients are demonstrated: cyberbullying victimization and perpetration is most common among students attending schools with weak leadership, and least common among students attending schools where teachers rate the leadership as strong. The relative difference is larger for cyberbullying perpetration than for cyberbullying victimization. Chi-square tests show that the associations are statistically significant.

To assess whether the association between teacher-rated school leadership and student-reported cyberbullying behavior is robust also when adjusting for student-level sociodemographic characteristics, multilevel analyses were performed. Results from two-level binary logistic regression models of cyberbullying victimization are presented in Table 3. Model 1 includes only student-level variables. Students who did not respond to the question on gender have an elevated risk of reporting cyberbullying victimization (OR 2.46, 95% CI 1.59–3.80). Also family structure demonstrates a statistically significant difference, indicating that students who do not live with two parents in the same household are more likely to report to be victims of cyberbullying compared to the reference category (OR 1.30, 95% CI 1.05–1.60). In Model 2, categories of teacher-rated school leadership are added. A graded association with cyberbullying victimization is demonstrated, although there is a statistically significant difference only between weak and strong school leadership. Students who attend schools with strong leadership are significantly less likely to report cyberbullying victimization compared to those attending schools where the teachers rate the leadership as weak (OR 0.69, 95% CI 0.51–0.94). Additional analyses showed that there is no statistically significant difference between intermediate and strong school leadership (data not presented). Furthermore, we also performed analyses where we included the continuous measure of school leadership instead of thirds. This measure did however not reach statistical significance (OR 0.98, 95% CI 0.95–1.01) (data not presented), indicating that using thirds of school leadership is a preferred strategy since it captures the non-linear association with cyberbullying victimization.

Results from analyses of cyberbullying perpetration are presented in Table 4. Model 1, containing only student-level variables, demonstrates that girls are less inclined than boys to be perpetrators of cyberbullying (OR 0.45, 95% CI 0.32–0.63). In addition, students with university educated parents are less likely to be engaged in cyberbullying perpetration (OR 0.71, 95% CI 0.52–0.98), whereas students who had lived in Sweden for less than 10 years are more likely to report cyberbullying perpetration compared to those who had lived in Sweden all their life (OR = 1.73, 95% CI 1.12–2.67). Model 2 adds school leadership. Also for cyberbullying perpetration, a graded association is demonstrated, although there is a statistically significant difference only between weak and strong school leadership. Students in schools where teachers rate the leadership as strong report less cyberbullying perpetration compared to students who attend schools where the leadership is rated as weak (OR 0.49, 95% CI 0.32–0.77). Additional analyses showed that the difference between intermediate and strong school leadership is significant only at the 10%-level (data not presented). Finally, we also conducted analyses of cyberbullying perpetration using the full index of school leadership. This was shown to be statistically significant at the 1%-level (OR 0.94, 95% CI 0.91–0.98) (data not presented).

## 4. Discussion

Cyberbullying is a significant problem among students, demonstrated not least by the fact that cyberbullying behavior is associated with adverse psychological health outcomes over and above involvement in traditional bullying behavior [4,8,10,11,12]. Hence, identifying preventive measures to combat cyberbullying is a relevant task. The aim of the present study was to examine whether teachers’ ratings of the school leadership is associated with cyberbullying victimization and perpetration among students. Our hypothesis that strong school leadership is associated with less cyberbullying behavior gained empirical support: results from the multilevel binary logistic regression analyses showed that, among students in schools characterized by relatively strong leadership, both cyberbullying victimization and perpetration is less common than among students attending schools where the leadership is relatively weak.

Our findings reflect those of two Norwegian studies that reported associations between teachers’ ratings of the schools’ professional culture in terms of leadership, teacher affiliation, and collaborative activities, and school-level means of bullying victimization and perpetration as reported by students [18,19]. These studies were however restricted to traditional bullying behavior. Our results also mirror those of a recent English study covering both traditional and cyberbullying behavior: Bevilacqua et al. [20] reported that among students in schools rated by the governmental Office for Standards in Education, Children’s Services and Skills (Ofsted) as suffering from poor quality, cyberbullying perpetration was more common than among students in schools with “Outstanding” quality. The authors underlined the significance of schools’ leadership and management in understanding the prevalence of bullying behavior [20]. The relevance of the quality of the school leadership and management for bullying victimization and perpetration also relate to the findings of Muijs [21], although differences in school policies, in particular those related to bullying, were shown to have greater explanatory power with regard to the between-school variance in bullying behavior [21].

How can then the association between school leadership and cyberbullying behavior be understood? A strong leadership directs the work for school improvement and can therefore be expected to be associated with less bullying behavior among students [19]. For instance, it seems reasonable to assume that schools with a strong management foster clarity of school rules, positive student-teacher relations, interventions against bullying, as well as strong collective efficacy, which are all school-contextual aspects that have been shown to be linked with less prevalence of traditional bullying behavior [13,14,15,16,17]. Another, related, interpretation of our results is that a strong leadership is a prerequisite for schools’ anti-bullying work to come into force and to be efficient. In Sweden, schools are required by law to work actively against bullying. All anti-bullying measures are however not equally ambitious or equally successful. As a conclusion from their evaluation of anti-bullying methods, The Swedish National Agency for Education [27] argued that to be successful, anti-bullying work needs to be embedded among all staff and students in the school. If this is the case, it does not seem far-fetched to assume that a strong leadership facilitates or may even be a prerequisite for the implementation of such an encompassing anti-bullying approach. It is also possible that other, non-observed school-contextual aspects are confounders or mediators in the association between school leadership and cyberbullying behavior, for instance school policies related to bullying [21]. A promising avenue for future research would be to disentangle the pathways and mechanisms between school leadership and cyberbullying behavior among students, exploring possible mediators. One potentially fruitful way of gaining a deeper understanding of the processes at work is to use statistical methods such as structural equation modelling; another way would be to apply qualitative methods and perform interviews with teachers and other school staff as well as with students. Additional relevant tasks for future inquiry are to investigate how other school organizational characteristics as well as school segregation are related to the prevalence of cyberbullying behavior among students.

While we found associations between school leadership and cyberbullying behavior, it should be underlined that there are also student-level characteristics that are associated with cyberbullying. For instance, students who do not live with two custodial parents in one household had an elevated risk of being cyberbullied, as well as students who did not answer the question on gender (possibly not wanting to define themselves as boys or girls). Accordingly, to gain a deeper understanding of cyberbullying, one should acknowledge the existence of social inequalities and power orders.

The main strength of the current study is the use of a new, large-scale data material that combines survey information from both teachers and students. The participation rate was reasonably high among both teachers and students. Our measure of school leadership was based on teachers’ responses to multiple items and our measures of cyberbullying behavior were based on survey responses from students, thus decreasing the risk of bias due to common method variance. Nevertheless, the study is also subject to limitations. As mentioned above, it is possible that there are other school-contextual aspects that may account for the association between school leadership and cyberbullying behavior among students. For instance, many Swedish schools use anti-bullying programs, but we lack information on the type and extent of practical anti-bullying work in the studied schools. Another limitation concerns the fact that both cyberbullying victimization and perpetration were captured by single items with dichotomous responses rather than by more elaborated measures. Furthermore, it is possible that the measure of cyberbullying perpetration had lower validity and reliability than that of cyberbullying victimization due to social desirability. On the other hand, the association between school leadership and cyberbullying perpetration was stronger than that between school leadership and cyberbullying victimization. This could theoretically be expected, since students who are subjected to cyberbullying victimization may be so by persons who do not attend the same school, and whose behavior is thus not affected by the school contextual characteristics of the school that the cyberbullying victim attends. Finally, it should be mentioned that since the data were collected among teachers and students in Stockholm, we cannot make generalizations to other contexts. Hence, further studies are needed to corroborate the findings.

## 5. Conclusions

Cyberbullying is a significant problem for those who are exposed, and thus, efforts to combat this type of bullying are needed. The school is one context which may act protectively against cyberbullying. Connecting school-level information based on teachers’ assessment of the school leadership with students’ reports on cyberbullying victimization and perpetration, the present study concludes that a strong school leadership is one potentially important component in the work against cyberbullying among students. A fruitful task for future inquiry is to investigate the relationship between school leadership and cyberbullying behavior in greater depth in order to discern the processes and mechanisms at work.

## Figures and Tables

**Table 1:** Descriptives of variables included in the analyses. *n*= 6067.

Descriptives	*n*	%
Cyberbullying victimization ^a^		
No	5246	92.7
Yes	411	7.3
Cyberbullying perpetration ^b^		
No	5607	97.0
Yes	174	3.0
Gender		
Boys	2798	46.1
Girls	3064	50.5
Missing information	205	3.4
Family structure		
Two parents in the same household	3777	62.3
Other	2290	37.7
Parents’ university education		
No parent	2068	34.1
At least one parent	3999	65.9
Migration background		
Lived in Sweden whole life	4937	81.4
Lived in Sweden ≥ 10 years	530	8.7
Lived in Sweden < 10 years	600	9.9
	Mean	s.d.
School leadership	34.23	4.24

^a^ *n* = 5657; ^b^ *n* = 5781.

**Table 2:** Description of categories of school leadership, and the occurrence of cyberbullying victimization and cyberbullying perpetration by categories of school leadership.

Categories of School Leadership	*n*_Students_	*n*_Schools_	School Leadership		Cyberbullying Victimization		Cyberbullying Perpetration	
			Mean	Range	%	Chi^2^	%	Chi^2^
School leadership								
Weak	2225	17	30.05	24.67–32.65	8.5		4.0	
Intermediate	1910	14	34.43	32.71–35.52	7.3		3.0	
Strong	1932	27	38.85	35.78–44.60	5.8	10.68 **	1.9	14.01 **

** *p* < 0.01.

**Table 3:** Results from two-level binary logistic regression models of cyberbullying victimization. Odds ratios and 95% confidence intervals. *n* = 5657 students within 58 schools.

Cyberbullying Victimization	Model 1		Model 2	
	OR	95% CI	OR	95% CI
**Student-level**				
Gender				
Boys (ref.)	1.00		1.00	
Girls	1.10	0.89–1.37	1.09	0.88–1.35
Missing information	2.46 ***	1.59–3.80	2.45 ***	1.59–3.79
Family structure				
Two parents in the same household (ref.)	1.00		1.00	
Other	1.30 *	1.05–1.60	1.30 *	1.05–1.60
Parents’ university education				
No parent (ref.)	1.00		1.00	
At least one parent	0.93	0.75–1.16	0.93	0.75–1.16
Migration background				
Lived in Sweden all life (ref.)	1.00		1.00	
Lived in Sweden ≥ 10 years	0.97	0.67–1.40	0.96	0.66–1.39
Lived in Sweden < 10 years	1.07	0.76–1.50	1.09	0.78–1.52
**School-level**				
School leadership				
Weak (ref.)			1.00	
Intermediate			0.85	0.62–1.15
Strong			0.69 *	0.51–0.94
School-level variance (s.e.)	0.08	(0.05)	0.05	(0.04)

*** *p* < 0.001; * *p* < 0.05.

**Table 4:** Results from two-level binary logistic regression models of cyberbullying perpetration. Odds ratios and 95% confidence intervals. *n* = 5781 students within 58 schools.

Cyberbullying Perpetration	Model 1		Model 2	
	OR	95% CI	OR	95% CI
**Student-level**				
Gender				
Boys (ref.)	1.00		1.00	
Girls	0.45 ***	0.32–0.63	0.45 ***	0.32–0.62
Missing information	0.73	0.31–1.69	0.73	0.31–1.69
Family structure				
Two parents in the same household (ref.)	1.00		1.00	
Other	1.22	0.89–1.67	1.21	0.89–1.66
Parents’ university education				
No parent (ref.)	1.00		1.00	
At least one parent	0.71 *	0.52–0.98	0.73	0.53–1.00
Migration background				
Lived in Sweden all life (ref.)	1.00		1.00	
Lived in Sweden ≥ 10 years	1.19	0.71–2.00	1.17	0.70–1.97
Lived in Sweden < 10 years	1.73 *	1.12–2.67	1.77 **	1.15–2.71
**School-level**				
School leadership				
Weak (ref.)			1.00	
Intermediate			0.75	0.51–1.11
Strong			0.49 **	0.32–0.77
School-level variance (s.e.)	0.10	(0.10)	0.04	(0.09)

*** *p* < 0.001; ** *p* < 0.01; * *p* < 0.05.
